# Novel Gammapapillomavirus type in the nasal cavity of a wild red colobus (Piliocolobus tephrosceles)

**DOI:** 10.1099/acmi.0.000866.v3

**Published:** 2024-08-20

**Authors:** Taylor E. Weary, Kavi P. M. Mehta, Tony L. Goldberg

**Affiliations:** 1 Department of Pathobiological Sciences, University of Wisconsin-Madison School of Veterinary Medicine, Madison, WI, USA; 2 Department of Comparative Biosciences, University of Wisconsin-Madison School of Veterinary Medicine, Madison, WI, USA

**Keywords:** E7, monkey, papillomavirus, primate, red colobus, Uganda

## Abstract

Papillomaviruses (PVs) are double-stranded, circular, epitheliotropic DNA viruses causing benign warts (papillomas) or inducing dysplasia that can progress to cancer. Although they have been identified in all vertebrate taxa, most classified types are human PVs (HPVs); relatively little is known about PVs in other species. Here we characterize a novel *Gammapapillomavirus* type, PtepPV1, from a nasal swab of a wild red colobus (*Piliocolobus tephrosceles*) in Kibale National Park, Uganda. The virus has a genome of 6576 bases, encoding the seven canonical early (E) ORFs (E6, E7, E1, E2, E4, E1^E4 and E8^E2) and two late (L) ORFs (L1 and L2) of the gammapapillomaviruses, and is 81.0% similar to HPV-mSK_118, detected in a cutaneous wart from an immunocompromised human patient, in the L1 gene at the amino acid level. Alphapapillomaviruses (genus *Alphapapillomavirus*) cause anogenital carcinomas such as cervical cancer and have been described previously in several nonhuman primates. However, the first gammapapillomavirus (genus *Gammapapillomavirus*), which cause transient cutaneous infections, was not described until 2019 in a healthy rhesus macaque (*Macaca mulatta*) genital swab. The new virus from red colobus, PtepPV1, has many genomic features encoded by high-risk oncogenic PVs, such as the E7 gene LXSXE and CXXC motifs, suggesting potential for pRb and zinc-finger binding, respectively. To our knowledge, PtepPV1 is also the first reported nonhuman primate PV found in the nasal cavity. PtepPV1 expands the known host range, geographical distribution, tissue tropism and biological characteristics of nonhuman primate PVs.

## Data Summary

The data that support these findings are openly available in NCBI GenBank (accession nos. ON745305, ON792783 and ON792784).

## Introduction

Members of the family *Papillomaviridae* are non-enveloped, double-stranded, circular DNA viruses that have infected vertebrates for at least 400 million years [1]. The papillomaviruses are currently classified into over 50 genera based on sequence identity across the L1 major capsid ORF [2]. The approximately 6–8 kb genome of papillomaviruses (PVs) is organized into three major regions, including an upstream regulatory region (URR) containing promoter sequences, early (E) nonstructural genes involved mainly in viral replication and a late (L) gene region which codes for two capsid proteins (L1 and L2) that self-assemble to encapsidate the genome for virion release. Most PVs cause asymptomatic, transient infections of the skin and mucous membranes and are part of the normal microbiome [3]. Entering basal cells through minor trauma, PVs can sometimes cause benign cutaneous warts on the skin (papillomas) and genitals (condyloma acuminata), with transmission occurring most often through direct contact and sexual activity, respectively [4]. However, some human papillomaviruses (HPVs) can persistently infect differentiated keratinocytes and progress to malignancy [3]. The E5 (if present), E6 and E7 proteins in oncogenic PVs are implicated in the onset of cancer by downregulating the p53, p21 and cyclin D1 cell-cycle regulators, which are important tumour suppressor pathways [5]. Indeed, infections with certain high-risk genital HPVs (all members of the genus *Alphapapillomavirus*) cause nearly all cases of cervical cancer worldwide, in addition to cancers of the vagina, vulva, penis, anus and oropharynx [6].

Black-and-white colobuses (*Colobus polykomus* and *C. guereza*) are a useful natural animal model of human cutaneous and venereal papillomatoses because lesions occur spontaneously in these species. Cutaneous papillomas were first identified on the palmar and plantar surfaces and dorsal digital surfaces of a *C. polykomos* housed at the St. Louis Zoo around 1970 [7], although a specific aetiological agent could not be confirmed at the time. Papillomas on the hands and feet were found in a laboratory-housed *C. guereza* a few years later [8], and a venereal papilloma was diagnosed on another *C. guereza* at the Brookfield Zoo in Chicago in 1987 [9]. The venereal papillomavirus was closely related to the *Alphapapillomavirus* HPV11, which also causes genital warts in humans, and was named CgPV1 (for *C. guereza* papillomavirus 1). Kloster *et al*. [10] obtained biopsies of the cutaneous papillomas of the monkey from Rangan *et al*.’s study as well as from a laryngeal carcinoma that developed afterwards in the same animal, and they were able to identify both lesions as papillomavirus-induced, although the virus was sufficiently different from CgPV1 to designate it CgPV2 (now categorized as a *Betapapillomavirus*).

Here we describe the identification and genomic characterization of a novel *Gammapapillomavirus* type from a wild adult male red colobus (*Piliocolobus tephrosceles*) from Kibale National Park, Uganda. To our knowledge, this virus is the first described gammapapillomavirus infecting a wild colobus and the first nonhuman primate (NHP) papillomavirus found in the nasal cavity. Although the monkey did not have any signs of warts or systemic illness at the time of sample collection, the virus also contains genomic features encoded by high-risk oncogenic PVs. Our results expand the host range, geographical distribution and biological characteristics of NHP papillomaviruses.

## Methods

### Field site

Kibale National Park, Uganda (795 km^2^; 0° 13′ to 0° 41′ N, 30° 19′ to 30° 32′ E) is notable for its high species diversity and biomass of primates, including the world’s densest population of red colobus [11,12]. Kibale’s red colobus population has been studied almost continuously since 1970 [13,14]. *Piliocolobus* species are currently the most threatened group of African monkeys according to the International Union for Conservation of Nature [15], and conservation efforts have been hindered by their particularly unstable taxonomy and the current inability to house them in captivity in wildlife sanctuaries or zoos [16].

### Specimen collection and preparation

Between January 2010 and June 2012, free-ranging monkeys of various species were sampled in Kibale as previously described [17] as part of a long-term study of animal and human health in the area [17,20]. A sterile Dacron swab was inserted into both nostrils of each immobilized animal and placed in 500 µl RNAlater nucleic acid preservation buffer (Thermo Fisher). In the field, samples were kept in liquid nitrogen then shipped to the USA in IATA-approved dry shippers that maintained the cold chain throughout until samples were kept in a −80 °C freezer for long-term storage.

### Metagenomic sequencing

To identify viruses, metagenomic sequencing was applied to nasal swabs of 16 animals, including three olive baboons (*Papio anubis*), two blue monkeys (*Cercopithecus mitis*), two black-and-white colobus (*C. guereza*), one L’Hoest’s monkey (*Cercopithecus lhoesti*), two gray-cheeked mangabeys (*Lophocebus albigena*), two red-tailed monkeys (*Cercopithecus ascanius*) and three red colobus, using previously described methods [17,21,23]. The tips of the Dacron swabs were homogenized in 150 µl RNAlater and 850 µl Hanks’ balanced salt solution (HBSS) and subjected to centrifuge clarification. Nucleases were applied to the supernatant to digest un-encapsidated nucleic acids [24]. The QIamp MinElute Virus Spin Kit (Qiagen) was employed to extract nucleic acids according to the manufacturer’s protocol but omitting carrier RNA. Then, cDNA was synthesized using the Superscript IV kit (Thermo Fisher) and random hexamer priming and purified with Agencourt AMPure XP beads (Beckman Coulter) as described previously [25,28]. The Illumina Nextera XT kit (Illumina) was employed to produce genomic libraries, which were sequenced on an Illumina MiSeq using 300×300 cycle (V3) paired-end chemistry. Two blank samples (a sterile Dacron swab in 500 µl RNAlater) were prepared and sequenced alongside the experimental samples as negative controls.

### Bioinformatics

We trimmed low-quality (Phred score <30) and short (<50 bp) sequences and discarded sequences matching known reagent contaminants or host DNA with CLC Genomics Workbench v.20.0.4 (Qiagen). We then subjected the remaining reads to *de novo* assembly using the metaviral option in SPAdes v.3.15.2 [29]. We compared the resulting contiguous sequences (contigs) to viruses in NCBI GenBank at both the nucleic acid and amino acid levels using BLASTn and BLASTx, respectively [30,31]. We retained contigs matching mammalian viruses, whereas contigs matching viruses of known nonmammalian hosts (e.g. insects, plants, fungi and phage) were discarded. The complete PV genome, including ORFs and putative functional motifs, was compared to all PV sequences currently uploaded to the Papillomavirus Episteme (PaVE) database [32] and annotated using PuMA [33].

### PV diagnostic PCR

To confirm PV infection, PCR primers were designed based on the PtepPV1 consensus sequence at the E6/E7 gene region, which has been used previously for HPV identification and typing [34]. DNA was extracted from the original nasal swab homogenate using the DNeasy Blood and Tissue Kit (Qiagen) according to the manufacturer's instructions. PCRs were carried out in 25 µl reactions containing 0.2 µM of each primer (forward: 5′-TGAGCAGGGAAATTCCCTAG-3′; reverse: 5′-CTGTTGCAGTGCTCCTAAGT-3′), 12.5 µl Qiagen 2× HotStarTaq DNA Polymerase, and 2 µl of template DNA on a C-1000 thermocycler (BioRad) with the following cycling conditions: 95 °C for 15 min; 45 cycles of 94 °C for 30 s, 50 °C for 30 s and 72 °C for 1 min; and 72 °C for 10 min.

Products approximately 650 bp in length were electrophoresed on a 2% agarose gel with ethidium bromide and 1 kb plus DNA length standards (New England Biolabs), visualized under UV light and photographed using a GelDoc XR imager (BioRad). Amplicons were excised from gels and purified with the Zymoclean Gel DNA Recovery Kit (Zymo Research), eluted in 6 µl elution buffer and directly sequenced on both strands on an ABI 3130xl Genetic Analyzer (Applied Biosystems) at the University of Wisconsin-Madison Biotechnology Center. Chromatograms 610 bases in length were proofread and assembled using Sequencher 4.10.1 (Gene Codes).

### Phylogenetic analysis

Phylogenetic relationships among related PVs were inferred from nucleotide sequences of the L1 capsid gene ORF, the most conserved region of the PV genome [32]. The consensus L1 nucleotide sequence of the PtepPV1 virus was first aligned with the L1 sequences of closely related NHP and human gammapapillomavirus types on PaVE [32] using muscle [35]. A maximum likelihood phylogenetic tree was reconstructed from the 222-position alignment using PhyML [36] and SMS [37] (K80+i model of molecular evolution) with 1000 bootstrap replicates and was displayed using FigTree 1.4.4 [38]. For further comparison of PtepPV1 with the phylogenetically closest HPV type (HPV-mSK_118, GenBank accession no. MH777260), pairwise similarities between the complete nucleotide/amino acid sequences of individual genes/proteins were calculated using the EMBOSS Water Pairwise Sequence Alignment tool [39].

## Results

### Virus identification and genomic characterization

After quality trimming and *in silico* subtraction of sequences associated with hosts and reagents, we retained a total of 1 987 302 sequence reads of average length 176 bp for analysis. We successfully assembled one complete circular PV genome of 6576 bp from a sample from an adult male red colobus (average coverage of 63×) and confirmed the presence of the virus by PCR. In accordance with naming conventions for the Animal Papillomavirus Reference Center [40], we have named this virus PtepPV1 (for *Piliocolobus tephrosceles* papillomavirus 1) (GenBank accession no. ON745305).

In addition to this virus, we identified two torque teno viruses (TTVs; family: *Anelloviridae*) in the nasal swab of a geriatric female red colobus (GenBank accession nos. ON792783 and ON792784). Although it is unclear whether TTVs are associated with disease in any species, respiratory secretions appear to be a primary route of infection, and they have been detected previously in nasopharyngeal swabs of children [41]. In humans, increased TTV viral load is associated with both immune suppression and ageing, which may be mediated by declining immune function with age [42]. Identifying these TTVs in a geriatric individual suggests this age-related pattern may exist in NHPs as well. No other eukaryotic viruses were detected in any of the other monkey nasal swabs.

**Fig. 1.**
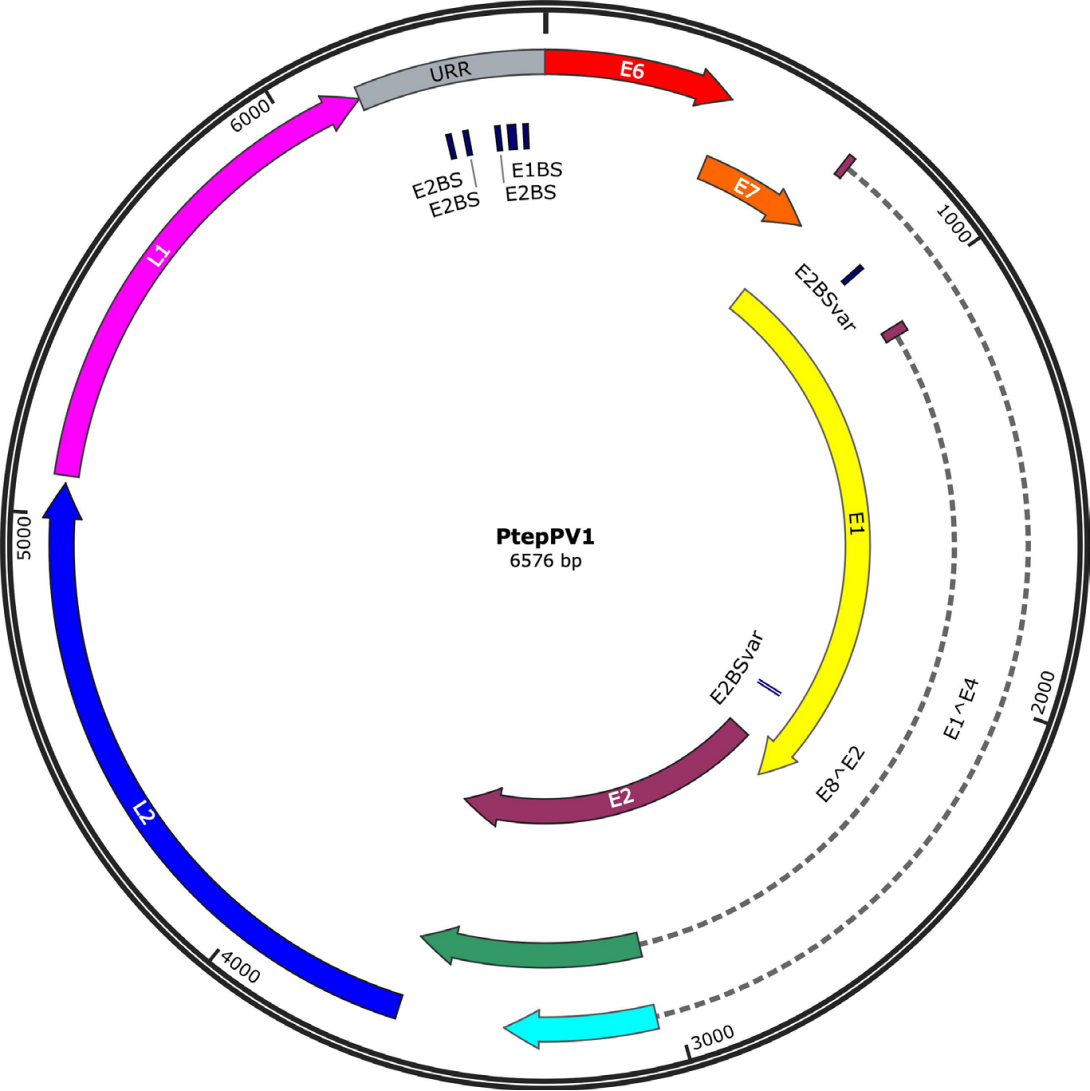
Map of the complete PtepPV1 genome. All positions were labelled from the first nucleotide of the E6 ORF. URR: upstream regulatory region, E2BS: E2 binding site, E2BSvar: E2 binding site variant, E1BS: E1 binding site. The map was created using SnapGene 6.0.5 (GSL Biotech LLC).

PtepPV1 has a circular genome of 6 576 bp with a guanine–cytosine (GC) content of 38.7%. The genome contains the seven canonical early (E) ORFs (E6, E7, E1, E2, E4, E1^E4 and E8^E2) and two late (L) ORFs (L1 and L2) of the gammapapillomaviruses, and it lacks the oncogenic E5 gene, consistent with the genome architecture of other members of the genus *Gammapapillomavirus* (Fig. 1). Nucleotide positions of all ORFs and putative functional motifs are listed in Table S1, available in the online version of this article.

The URR, between the 3′ end of L1 and the 5′ end of E6, contains the origin of replication and promoter sequences that orchestrate the successive transcription of early and late genes for viral replication first in actively dividing basal cells and then virion assembly and release from differentiated keratinocytes [43]. The URR of PtepPV1 is 412 bp long and contains two TATA box (TATAA) promoter sequences. Four E2 binding sites (ACC-X_6_-GGT), which are necessary for DNA replication, were identified in the URR, while two additional E2 binding site variants (ACC-X_6_-GTT) present in gammapapillomaviruses [44] were found in the E1 ORF. There is an E1 binding site (TGATTGCTGACAACTATCAT) in the URR, unlike in other recently described NHP gammapapillomaviruses (CpenPV1 and CpenPV2 in the black-tufted marmoset, *Callithrix penicillata*) [45], which indicates that PtepPV1 contains more than the minimum origin of replication necessary for its genus, potentially enhancing replication initiation by *trans*-acting viral elements, rather than host cellular machinery [46]. The PtepPV1 genome contains 12 putative polyadenylation sites (AATAAA) for processing late viral mRNA transcripts.

### Phylogenetic analysis

The complete L1 ORF shares 75.3% nucleotide and 81.0% amino acid identity with the closest known gammapapillomavirus, HPV-mSK_118, an unclassified PV type from a skin swab of a human patient with a rare primary immunodeficiency in the USA (GenBank accession no. MH777260). The other PtepPV1 ORFs vary in similarity to those of HPV-mSK_118 to a minimum of 51.9% amino acid identity (Table 1). Of the current PV reference genomes [2], PtepPV1 is most closely related to gammapapillomavirus 14 (HPV131, GenBank accession no. NC_014954, 72.9% nucleotide and 74.9% amino acid identity for L1). The L1 ORF nucleotide sequence differs by ≥10% from other known gammapapillomaviruses but is <30% dissimilar, indicating that this virus fulfils ICTV criteria to be classified as a new *Gammapapillomavirus* type, but not a new species [47]. In support of this classification, the virus was found in a novel animal host, the Ugandan red colobus.

**Fig. 2.**
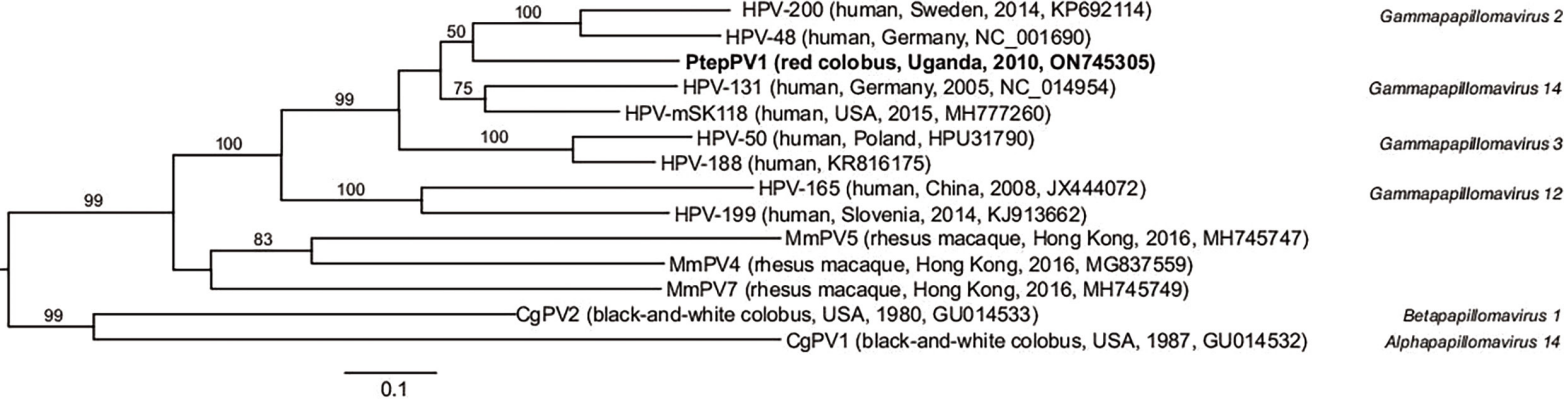
Maximum likelihood phylogenetic tree of gammapapillomavirus types closely related to PtepPV1 (bold type) based on a 222-position alignment of L1 (major capsid) gene sequences. Virus names are followed by (host, location, year, GenBank accession number). The tree is outgroup rooted with the other two known colobus PVs (CgPV1 and CgPV2). Bootstrap values ≥50% are represented by numbers beside branches (1000 replicates). Bar, 0.1 nt substitutions per site.

PtepPV1 is more genetically similar to human gammapapillomaviruses than either NHP gammapapillomaviruses detected in rhesus macaques or the other known colobus PVs (Fig. 2).

**Table 1.** Comparison of open reading frames between PtepPV1 and the phylogenetically most closely related PV types, HPV-mSK_118 and HPV131 (aa=amino acids, nt = nucleotides)

	Length (aa)			Percentage identity (aa, nt)		
ORF	PtepPV1	HPV-mSK_118	HPV131	PtepPV1/HPV-mSK_118	PtepPV1/HPV131	HPV-mSK_118/HPV131
E6	139	141	141	51.9, 65.2	54.2, 62.3	63.8, 70.2
E7	98	93	94	62.9, 64.0	59.8, 60.1	78.7, 84.6
E1	603	660	663	65.6, 71.9	68.8, 72.2	74.4, 76.0
E2	394	395	395	62.8, 70.4	62.1, 69.7	75.0, 79.2
L2	486	502	511	62.0, 65.3	68.7, 67.2	70.8, 71.3
L1	360	518	513	81.0, 73.8	74.9, 71.4	83.3, 76.5
E1^E4	116	117	114	55.4, 66.7	56.4, 66.2	64.0, 78.4
E8^E2	197	198	198	55.2, 68.5	56.2, 66.3	73.4, 78.7

## Discussion

Here we describe a papillomavirus type (PtepPV1) of the genus *Gammapapillomavirus* from the nares of a Ugandan red colobus. Detection of PtepPV1 in the Ugandan red colobus expands the known host range, geographical distribution and diversity of NHP PVs. Red colobuses are separated from black-and-white colobuses by 7.5 million years of evolution [48], much more recently than the radiation of primate PVs (~41 million years ago), which coincides with the time Old World and New World monkeys diverged [49]. The known black-and-white colobus PVs (CgPV1 and CgPV2) are alpha- and betapapillomaviruses, respectively [7,8], but the closest related PVs to PtepPV1 are HPVs recovered from the cutaneous warts of immunocompromised patients. This observation underscores the PV tendency to cluster phylogenetically by niche adaptation (i.e. different types of epithelial cells) rather than by host species [50]. In addition to black-and-white colobuses, macaques [51,52] and olive baboons [53] are the only Old World monkeys (cercopithecines) known to harbour PVs of any genus. In cercopithecines, gammapapillomaviruses have previously only been detected in macaques [51].

PtepPV1 contains genomic features similar to related gammapapillomaviruses but with more binding sites for viral replication machinery despite a smaller genome size. The virus has a truncated E1 (DNA helicase) and L1 (major capsid) compared to the closely related HPV-mSK_118 and HPV131. Compared to these other PV types, PtepPV1’s most similar gene is L1 while its least similar gene is E6 (an oncogene involved in inactivation of the pro-apoptotic p53 pathway). PtepPV1 contains more E2 binding sites than either related virus (four plus two E2 binding site variants versus two in HPV-mSK_118 and three in HPV131), suggesting it may have an increased ability to initiate DNA replication and establish persistent infections in actively dividing cells. The E1 binding site in the URR also promotes DNA replication by *trans*-acting viral elements, suggesting PtepPV1 may have increased replicative efficiency similar to the oncogenic HPV16 or bovine BPV1 [54].

The presence of E7, an accessory oncogene not encoded by all PVs, in PtepPV1 is noteworthy for several reasons. The immortalization and pro-proliferative capacity of E7s vary substantially between PVs which contain them due to differences in domains that degrade the cell-cycle regulator pRb and disrupt the DREAM complex [55,56]. Higher affinity for pRb binding by E7 is predictive of oncogenic potential and immortalization frequency [57]. E7s can be generally divided into three domains known as conserved regions 1, 2 and 3 (CR1, CR2 and CR3). CR1 shares homology with both SV40T antigen and adenovirus E1A [58]. The CR2 domain in high-risk oncogenic PVs often contains an LXCXE motif that is important for binding pRb, P130 and p105 that regulate E2F transcription factor cell cycle regulation. CR2 can also encode a CKII phosphorylation target which can be important for cellular immortalization [59]. E7 CR3 can contain two CXXC motifs (29 aa apart) deriving a zinc-finger binding domain that contribute to cellular transformation capacity and E7 protein stabilization [60,61]. Studies also indicate that specific residues in E7 CR3s can contribute to pRb binding and high-order E7 structures (oligomers) [62].

Interestingly, PtepPV1 E7 contains many of these characteristics encoded by pro-proliferative PVs. PtepPV1 CR2 contains an LXSXE motif instead of the LXCXE motif observed in high-risk oncogenic HPVs. Studies suggest that although LXCXE is a powerful pRb binder, LXSXE not only is important for pRb and DREAM complex disruption in multiple contexts but is also indispensable for pRb degradation in oncogenic canine papilloma virus 2 (CPV2), an oncogenic and epidermotropic virus primarily found in immunosuppressed animals [63,64]. PtepPV1 CR2 also encodes a putative CKII site (amino acids 29–32) proximal to the LXSXE domain, similar to those observed in PVs with transformation potential. Finally, PtepPV1 CR3 encodes two CXXC motifs (29 aa apart) indicating zinc-finger binding potential. These features suggest wider and potential pro-proliferative and neoplastic potential. No similar high-risk features were identified in any other PtepPV1 protein. Future studies will be important to define this PV’s potential (or lack thereof) for papillomatosis or neoplastic events in primates and other hosts.

HPVs found in the human nasal cavity can cause recurrent respiratory papillomatosis in children and adults [4] and are thought to be the causative agent of over 50% of head and neck cancers [65]. However, no PVs to date have been found in the NHP nasal cavity, to our knowledge. Previous cutaneous alpha- and betapapillomaviruses in *C. guereza*, producing papillomas on fingers and toes, were discovered in the 1980s and revisited in 2011 [8,66]. PtepPV1 and other PVs yet to be discovered may have the potential to induce both papillomatosis and/or neoplasia, although these disease states have not been reported. Identification and characterization of other novel PVs across NHPs and other species, especially those infecting the nasal mucosa, would undoubtedly shed light on the range of pathogenic potential of such viruses. Future *in vitro* replication studies are necessary to confirm the presence and function of molecular domains identified *in silico* in the current study. Similarly, such studies would help elucidate the true tissue tropism of PtepPV1 and similar viruses, given that PtepPV1 could have been detected in the nasal cavity opportunistically (e.g. transferred from the skin or oral mucosa via saliva during grooming). NHP PVs have been detected in saliva samples from chimpanzees (*Pan troglodytes*) [67] and lemurs (*Varecia variegata* and *V. rubra*) [68], raising the possibility of noninvasive sampling for NHP PVs [69,70]. Of note, true dual tissue tropism of the nasopharynx and anogenital region has been demonstrated for HPV199, another gammapapillomavirus [71].

Common predisposing factors for PV infections include primary or secondary immunodeficiencies in addition to a history of receiving an organ transplant or dialysis treatment in humans [72]. A thorough physical exam of this red colobus by two veterinarians at the time of sample collection showed no apparent clinical signs of immunocompromise, systemic illness or warts, such as in the black-and-white colobuses. However, blood samples collected from this same individual in 2006 and 2010 tested positive for several other novel viruses, including three retroviruses [20], two simian arteriviruses [17] and a simian pegivirus [21], and were seroreactive for an unknown orthopoxvirus [73]. Further research is needed to determine whether co-infection might modify the pathogenesis of PtepPV1, although this individual has remained healthy and is by no means an outlier with respect to the diversity or loads of viruses it carried [21,74].

Black-and-white colobus have been a useful natural animal model of human cutaneous and venereal papillomatoses because their PV infections and dermatoses occur spontaneously [7,9, 75]. The discovery of PtepPV1 in a red colobus expands our knowledge of the genome characteristics of NHP papillomaviruses. Should this virus be isolated in the future, it may similarly expand our knowledge of the biology and clinical characteristics of PVs in NHPs.

## Abbreviations

BPV1: bovine papillomavirus type 1
CgPV1: Colobus guereza papillomavirus 1
E: early
HBSS: Hanks' balanced salt solution
HPV: human papillomavirus
L: late
NHP: nonhuman primate
ORF: open reading frame
PaVE: Papillomavirus Episteme
PCR: polymerase chain reaction
PtepPV1: Novel papillomavirus from wild red colobus in Uganda
PV: papillomavirus
PVs: papillomaviruses
TTV: torque teno virus
URR: upstream regulatory region
